# Exercise Effects on Left Ventricular Remodeling in Patients with Cardiometabolic Risk Factors

**DOI:** 10.3390/life13081742

**Published:** 2023-08-14

**Authors:** Evanthia Bletsa, Evangelos Oikonomou, Kyriakos Dimitriadis, Panagiota K. Stampouloglou, Christos Fragoulis, Stavroula P. Lontou, Emmanouil Korakas, Eirini Beneki, Konstantinos Kalogeras, Vaia Lambadiari, Konstantinos Tsioufis, Manolis Vavouranakis, Gerasimos Siasos

**Affiliations:** 1 3rd Department of Cardiology, National and Kapodistrian University of Athens, Medical School, Sotiria Chest Disease Hospital, 11527 Athens, Greece; evabletsa@gmail.com (E.B.); stampoulogloupanagiota@gmail.com (P.K.S.); kalogerask@yahoo.gr (K.K.); vavouran@med.uoa.gr (M.V.); gsiasos@med.uoa.gr (G.S.); 2 Cardiometabolic Disease Unit, 3rd Department of Cardiology, National and Kapodistrian University of Athens, Medical School, Sotiria Chest Disease Hospital, 11527 Athens, Greece; mankor-th@hotmail.com (E.K.); vlambadiari@gmail.com (V.L.); 3 1st Department of Cardiology, National and Kapodistrian University of Athens, Medical School, Hippokrateion General Hospital, 11527 Athens, Greece; dimitriadiskyr@yahoo.gr (K.D.); christosfragoulis@yahoo.com (C.F.); e.beneki@hotmail.com (E.B.); ktsioufis@gmail.com (K.T.); 4 Heart and Diabetes Center, National and Kapodistrian University of Athens, Medical School, Hippokrateion General Hospital, 11527 Athens, Greece; slondou@gmail.com; 5 2nd Department of Internal Medicine, Medical School, National and Kapodistrian University of Athens, Attikon University Hospital, 12462 Athens, Greece

**Keywords:** exercise, diabetes, obesity, hypertension, ventricular remodeling, heart failure

## Abstract

Left ventricular (LV) remodeling is a dynamic process, which is characterized by changes in ventricular size, shape, and wall thickness, thus altering myocardial geometry and function, and is considered as a negative prognostic factor in patients with heart failure (HF). Hypertension, type 2 diabetes (T2D), and obesity are strongly correlated with the development and the progression of LV remodeling, LV hypertrophy, and LV systolic and/or diastolic dysfunction. Indeed, the beneficial impact of exercise training on primary and secondary prevention of cardiovascular disease (CVD) has been well-established. Recent studies have highlighted that exercise training enhances functional capacity, muscle strength and endurance, cardiac function, and cardiac-related biomarkers among patients with established coronary artery disease (CAD) or HF, thus substantially improving their cardiovascular prognosis, survival rates, and need for rehospitalization. Therefore, in this review article, we discuss the evidence of LV remodeling in patients with cardiometabolic risk factors, such as hypertension, T2D, and obesity, and also highlight the current studies evaluating the effect of exercise training on LV remodeling in these patients.

## 1. Introduction

It is estimated that more than 26 million adults suffer from heart failure (HF) worldwide, with the prevalence rates growing rapidly [1]. According to the current literature, 35–50% of patients with HF experience frequent rehospitalizations within 6 months of discharge, thus deteriorating their prognosis and incurring billions in costs [2]. Left ventricular hypertrophy (LVH), characterized by an increased left ventricular (LV) mass and cardiomyocyte hypertrophy, mainly increases the cardiovascular risk. Hypertension, type 2 diabetes (T2D), chronic kidney disease, and aortic stenosis are considered major risk factors for LVH [3]. Furthermore, LVH has been associated with an increased risk for LV dysfunction, HF, arrythmias, stroke, and sudden cardiac death [4].

**Figure 1:**
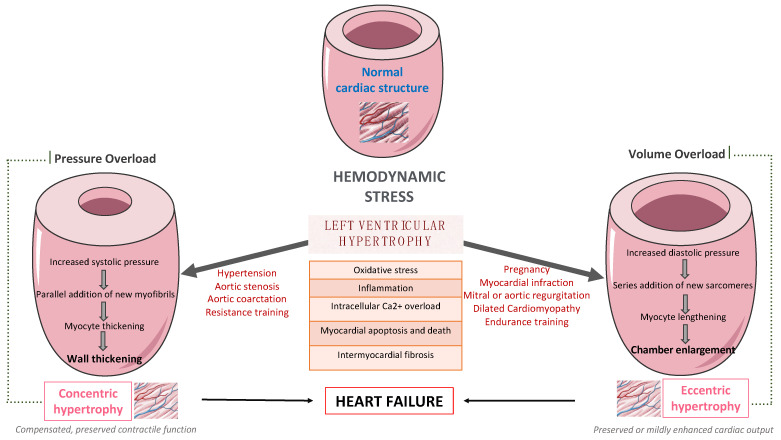
The pathway from left ventricular hypertrophy to heart failure.

LV remodeling is a dynamic process, which is characterized by changes in ventricular size, shape, and wall thickness, thus altering myocardial geometry and function [5]. It is considered as a negative prognostic factor in patients with HF. More specifically, obesity and hypertension cause an increase in systemic pressure, afterload, and wall stress, thus leading to the development of LVH [6]. Up to 60% of patients with hypertension may present with signs of an increased LV mass on echocardiography. Mild to moderate hypertension and LVH are commonly accompanied by varying degrees of impaired LV diastolic filling with normal or mild increased systolic performance at rest, as well as diminished coronary vasodilator capacity. Nevertheless, LVH might evolve to overt systolic and diastolic dysfunction, thus leading to the development and progression of heart failure with reduced eject fraction (HFrEF) or heart failure with preserved eject fraction (HFpEF), respectively, as presented in Figure 1.

Many complex and multifactorial mechanisms resulting in transcriptional, signaling, structural, and electrophysiological changes are involved in this process of LV remodeling. More specifically, oxidative stress and altered intracellular calcium metabolism provoke cardiomyocyte hypertrophy, thus resulting in impaired contraction and relaxation, as well as an increased risk for fatal ventricular arrythmias and sudden cardiac death [7]. Of note, interstitial and replacement fibrosis play a pivotal role in the progression of LV remodeling.

On the one hand, LV remodeling predicts adverse clinical outcomes, and possible regression seems to limit them, thus improving patient prognosis. On the other hand, short-term improvement in LV remodeling mediated by novel pharmaceutical agents and medical devices is associated with long-term improvement of clinical outcomes among patients with LV dysfunction [8]. More specifically, empagliflozin, a sodium–glucose co-transporter-2 inhibitor (SGLT-2i), ameliorates LV remodeling at 6 months [9] and cardiovascular outcomes after 2 years of treatment [10]. Similarly, sacubitril–valsartan, an angiotensin receptor–neprilysin inhibitor (ARNI), improves LV remodeling at 12 months [11] and clinical outcomes after 2 years of treatment [12]. Furthermore, the beneficial impact of exercise training on primary and secondary prevention of many clinical conditions, such as cardiovascular disease (CVD), T2D, as well as obesity, has been well-established so far. Recent studies highlight that exercise training enhances functional capacity, muscle strength and endurance, cardiac function, and cardiac-related biomarkers [13] among patients with established coronary artery disease (CAD) [14] or HF [15], thus improving substantially their cardiovascular prognosis, survival rates, and need for rehospitalization.

In this review article, we discuss the evidence of LV remodeling among patients with cardiometabolic risk factors, such as hypertension, T2D, and obesity, and we also focus on current studies evaluating the effect of exercise training on LV remodeling in these patients.

## 2. LV Remodeling Pattern among Patients with Cardiometabolic Risk Factors

### 2.1. Athlete’s Heart

Exercise training typically increases heart rate and blood pressure, leading to specific cardiac changes among healthy individuals, especially highly trained athletes [16]. This condition should be differentiated from hypertension-induced cardiac maladaptation and LVH, thus it is further discussed below. “Athlete’s heart” is characterized by eccentric LVH, especially among elite athletes, and those participating in endurance and resistance training programs, whereas the related functional and structural changes occur parallel with exercise training, as an adaptation to intensive and chronic hemodynamic overload. Consequently, LVH might be a physiological adaptation to strenuous physical exercise, as observed in athletes, although it is generally benign. However, it strongly correlated with the type of exercise [17]. Dynamic exercise, such as running and swimming, causes volume overload, whereas static exercise, such a weightlifting, increases pressure load. Aerobic exercise programs induce normal cardiac remodeling characterized by increased right ventricular (RV) and LV chamber dimensions and wall thickness, increased left atrial (LA) cavity size along with normal systolic and diastolic function, although resistance training mildly increases LV thickness, with no effect on LV chamber size. The increase in LV wall thickness and LA cavity is even greater in master endurance athletes [18]. LV wall thickness may exceed the normal upper limits of 13 to 15 mm and up to 16 mm in elite male athletes. Of note, exercise programs combining both aerobic and anaerobic types of activities, such as prolonged cycling, rowing, and swimming, result in mixed structural and functional cardiac adaptations where athletes experience the most increase in LV mass [19].

Interestingly, athletes may have a 15–20% increase in LV wall thickness, a 10% increase in LV cavity size, and a 24% increase in RV cavity size. LV thickness up to 15 mm is commonly apparent in most athletes, while the most thickened region is the anterior portion of the ventricular septum [20]. Morphologic adaptation to training in athletes enlarges the cavity size to an end-diastolic diameter ≥55 mm [6]. Nevertheless, “athlete’s heart” differentiates from hypertension-induced cardiac maladaptation and LVH. The overall pattern of LVH in “athlete’s heart” seems to be symmetric and homogenous [21]. However, athletes with LVH do not have impaired left ventricular ejection fraction (LVEF) or any evidence of systolic or diastolic dysfunction. Therefore, chronic exercise-mediated cardiac adaptations observed in athletes are considered to be normal hemodynamic responses, which do not correlate with increased risk of diastolic or systolic dysfunction, arrythmias, or adverse prognosis, whereas they regress gradually, when there is exercise restriction. Moreover, cardiac dimensions, LV hypertrophy, and LA dilatation generally regress to normal values after a period of detraining and deconditioning, which can take up to several years in longtime athletes [22]. However, an increase in LV mass, LV cavity size, and LA diameter might persist in up to 50% of endurance athletes even after 3 years of detraining.

### 2.2. Hypertensive Heart Disease

Chronic hemodynamic overload in hypertension provokes major structural and functional changes leading to LVH. More specifically, chronically increased blood pressure can cause LV pressure overload, thus increasing the LV workload and resulting in LVH [6]. This clinical condition is characterized by hypertrophy of the existing cardiomyocytes, addition of sarcomeres, and increased deposition of collagen and extracellular matrix, leading to an increase in ventricular mass.

Typical echocardiographic structural findings, including concentric remodeling, concentric hypertrophy or even a combination of concentric and eccentric hypertrophy, along with increased LV wall thickness and LV diastolic and systolic dimensions, have been described. Interestingly, healthy individuals, who do not exceed blood pressure levels above 150 mmHg during exercise, do not stimulate cardiac remodeling, and as a result, they are less likely to develop LVH [6]. So far, the European Society of Hypertension along with European Society of Cardiology have published recommendations for LVH evaluation based on electrocardiogram and echocardiogram findings. Although the ECG is a low-cost and widely used method, echocardiogram is the most preferred method for assessing LV size and function [23].

### 2.3. Diabetic Cardiomyopathy

T2D is a systemic disease with detrimental macro- and micro-vascular complications. Patients with T2D have a 2.5-fold increased risk to develop HF independently of age or concomitant comorbidities, such as CAD and dyslipidemia, whereas these patients represent one third of the HF population in clinical trials [24]. The term “diabetic myocardiopathy” is a clinical condition characterized by impairments in cardiac structure and functions independently of the macrovascular complications from diabetes (including hypertension, CAD, and atherosclerosis) [25]. Of note, cardiac dysfunction commonly remains clinically silent and as a result underdiagnosed until the latest stages of the disease, whereas almost 50% of asymptomatic, normotensive patients with T2D and good glycemic control demonstrate a degree of cardiac dysfunction.

Diabetes-induced LV diastolic dysfunction caused by prolonged and delayed LV filling and relaxation is often presented in the absence of concomitant impairments in LV systolic function [26]. Moreover, diabetes induces abnormal collagen deposition, cardiomyocyte hypertrophy, and cardiomyocyte loss via myocardial cell death pathways resulting in cardiac fibrosis and hypertrophy. Furthermore, coronary microvascular hypoperfusion, as characterized by impairment in the coronary flow reserve and myocardial blood flow with increased coronary resistance, is common among patients with T2D. Complex pathophysiological pathways, including oxidative stress, inflammation, impaired Ca2+ metabolism, mitochondrial and metabolic dysfunction, endoplasmic reticulum stress, along with alterations to insulin sensitivity and signaling, gene regulation, neurohumoral activation, and cardiac cell death, seem to be the main contributors to the development and progress of diabetic cardiomyopathy [27].

### 2.4. Obesity-Related Cardiomyopathy

Obesity is a multifactorial metabolic disorder characterized by a heterogeneous complex of biological, socioeconomic, and environmental factors leading to adverse health outcomes [28]. According to recent epidemiological data, it is estimated that more than 603 million adults suffer already from obesity worldwide, whereas prevalence rates grow rapidly [29]. Obesity has been strongly correlated with the presence of T2D, hypertension, and various lipid disorders, such as triglyceridemia, low levels of high-density lipoprotein, increased levels of small dense low lipoprotein and apoprotein B, thus increasing the cardiovascular risk [30]. Especially, central, visceral obesity is a pivotal cardiovascular risk factor. Pericardial and epicardial adipose tissue seem to be also a main contributor for CVD [31]. Obesity-induced insulin resistance, hyperinsulinemia, endothelial dysfunction, lipid accumulation, chronic low-grade systematic inflammation, oxidative stress, and prothrombotic status seems to be the main pathophysiological pathways leading to the development and progression of CVD.

Obesity is associated with atherosclerosis, abnormalities in the coronary microvasculature, and as a result, an increased risk for CAD. Furthermore, excess adipose tissue accumulation leads to major hemodynamic changes, including plasma volume expansion increased blood pressure, cardiac output, as well as myocardial wall stress [32]. Ectopic pericardial and epicardial adipose tissue (EAT) induce myocardial fat accumulation, resulting in local inflammation, macrophage infiltration, and cytokine gene expression, thus leading to subsequent myocardial fibrosis and cardiomyocytes hypertrophy. Of note, concentric LV remodeling and LVH, right ventricular dilatation, and right ventricular dysfunction have been reported among patients with obesity [31]. More specifically, plasma volume expansion and EAT expansion provoke increased RV dilatation, greater pericardial restraint, and heightened ventricular interdependence, thus creating LV restriction and increased filling pressures. There is a specific obesity-related LV remodeling that might lead to an obese-related phenotype of HFpEF [33]. Considering that patients with obesity present impaired systolic and diastolic cardiac function, they are more likely to develop HF to remove it. Indeed, obese patients have a 56% higher risk of developing HFpEF [34]. Of note, there are promising data that SGLT-2is reduce EAT volume, thus improving LV remodeling [35,36].

## 3. The Effect of Exercise Training on Left Ventricular Remodeling among Patients with Cardiometabolic Risk Factors

### 3.1. In Patients with Hypertension

According to the current literature, exercise training leads to a substantial reduction in resting systolic and diastolic blood pressure, as well as in LVH among hypertensive patients [37,38], as presented in Table 1. Additionally, recent studies indicate that moderate and regular physical activity reduces significantly total peripheral resistance [39]. Exercise-mediated hemodynamic changes include also an increased cardiac output along with the redistribution of blood flow to muscular territories. So far, there is evidence that exercise training may reduce LV hypertrophy in parallel with systolic and diastolic blood pressure improvement [40].

Turner et al. were among the first to report that exercise training may induce regression of LVH and LV concentric remodeling among patients with mild or moderate hypertension [41]. Specifically, exercise training improved aerobic efficacy by 16% and decreased substantially systolic and diastolic blood pressure, LV wall thickness, as well as LV mass index. Of note, LVH regression was mainly attributed to the reduction in the systolic blood pressure. Indeed, exercise training improves significantly systolic and diastolic blood pressure among patients with mild or moderate hypertension.

Furthermore, low-fit individuals with hypertension seem to have a higher LV mass index when compared to the moderate and high-fit individuals [38]. In this randomized controlled trial, 16 weeks of aerobic exercise led to a substantial regression of LV mass and thickness of the interventricular septum, which were mainly attributed to a linear reduction in systolic and diastolic blood pressure [38]. Similarly, regular exercise training results in lowering blood pressure, LV mass index, as well as exercise capacity among patients with borderline or mild hypertension [40]. An exercise-mediated decreased posterior wall and an intraventricular septal thickness are also found in hypertensive patients [42]. Furthermore, there is also evidence that a 1-MET increase in workload offers a 42% reduction in the risk of LVH [43]. It is important to note that regular physical activity seems to prevent the development of LVH among hypertensive patients at stage 1 [44]. More specifically, patients in the physically active group were less likely to develop LVH when compared to those following a sedentary lifestyle, after a median follow-up of 8.3 years.

### 3.2. In Patients with Type 2 Diabetes

So far, there is evidence that exercise training may improve both LV systolic and diastolic function in patients with diabetes, resulting in favorable changes in stroke volume, LVEF, end-systolic volumes, as well as LV filling [45]. Both endurance and combined endurance and resistance exercise training positively affect cardiovascular parameters among patients with T2D [46]. According to a randomized clinical trial, high intensity intermittent training (HIIT) seems to improve substantially cardiac function and structure among patients with T2D, resulting finally in a positive cardiac remodeling [47]. In more details, this type of 12-month exercise interventional program ameliorated both LV mass and systolic function when compared to standard care. Of note, these changes were accompanied by modest improvements in glycemic control.

Furthermore, Otten et al. reported that supervised exercise training (3 weeks/hour) paired with a paleolithic diet (based on vegetables, fruits, berries, nuts, seafood, eggs, fish, and lean meat with a high restriction of dairy products, cereals, legumes, refined fats, added sugar and salt) resulted in favorable metabolic and cardiac changes with a decrease in triglycerides levels and LV mass to end-diastolic volume ratio and an increase in LVEDV and stroke volume, among overweight and obese patients with T2D [48]. Similarly, exercise training seems to also improve diastolic function among patients with T2D. In a randomized clinical trial, a 12-week supervised aerobic exercise training program improved diastolic function in the absence of any major effects on LV remodeling, perfusion, or aortic stiffening, among asymptomatic young patients with T2D [49]. Interestingly, exercise-mediated favorable changes in a LV remodeling index seem to be the best predictor of improvement in LV diastolic function after the lifestyle intervention program, including increased physical activity among patients with T2D and CAD [50].

### 3.3. In patients with Type Obesity

Physical activity improves LVH among patients with obesity and hypertension. In more details, higher physical activity was associated with a reduction in the LV mass index and an improvement in LVH, as well as cardiac biomarkers, such as N-terminal pro-atrial natriuretic peptide (NT-pro BNP) and a mid-regional sequence of pro-A-type natriuretic peptide (MR pro-ANP) [51]. Similarly, a decrease in triglycerides levels and LV mass to end-diastolic volume ratio and an increase in LVEDV and stroke volume are reported among overweight and obese patients with T2D who participated in supervised exercise training programs [48]. Of note, the beneficial effects of exercise training on the reduction in LV mass are apparent regardless of whether the obese patients are normotensive or hypertensive. More specifically, Himeno et al. found that mild exercise together with mild hypocaloric intake resulted in significant weight and LV mass reduction among obese patients, after a 12-week intervention period [52]. Indeed, LV mass was significantly decreased among obese patients regardless of the presence of hypertension or not, whereas significant changes were found in the systolic, diastolic, and mean blood pressure. These data provide evidence that an exercise-mediated reduction in LV mass is not only attributed to a reduction in blood pressure and weight loss, but also to further mechanisms, such as improved cardiac autonomous function [53], myocardial metabolism and metabolic flexibility, as well as reduced LV stiffness [54].

Main studies evaluating the effect of exercise training on LV remodeling in patients with cardiometabolic risk factors, such as hypertension, T2D, and obesity, are summarized below in Table 1.

**Table 1:** Main studies evaluating the effect of exercise training on LV remodeling in patients with cardiometabolic risk factors, such as hypertension, type 2 diabetes, and obesity.

Author	Type of Study	Patient Characteristics	Main Findings
Zanettini et al. [37]	Prospective cohort study	14 sedentary patients with untreated diastolic BP (90–104 mmHg) 12-week supervised exercise program	Exercise-mediated increase in aerobic fitness significantly reduced resting systolic and diastolic BP, mean systolic and diastolic 24 h BP, as well as LV mass index.
Kokkinos et al. [38]	Randomized controlled trial	46 male patients with severe hypertension 35–76 years of age 16- or 32-week exercise program plus antihypertensive medication or antihypertensive medication alone	Diastolic BP decreased in the patients who exercised, whereas it increased slightly in those who did not exercise. Thickness of interventricular septum, LV mass, and mass index decreased significantly only in the patients who exercised.
Turner et al. [41]	Prospective cohort study	11 patients with mild to moderate hypertension vs. 7 sedentary hypertensive patients as controls 65.5 ± 1.2 vs. 68.5 ± 1 years of age 6.8 ± 3.8 months of an exercise program	Exercise training decreased systolic and diastolic BP, LV wall thickness and mass, as well as wall thickness to radius. Only the reduction in resting systolic BP was correlated significantly with the regression of concentric remodeling.
Pitsavos et al. [40]	Randomized controlled trial	40 patients with borderline to mild hypertension 53 ± 7 years of age 16-week exercise aerobic program or standard care	Systolic and diastolic BP, as well as heart rate were significantly lower in the exercise group compared to the control group. LV mass index decreased significantly only in the exercise group.
Palatini et al. [44]	Prospective cohort study	454 patients with stage 1 hypertension 33.1 ± 8.4 years of age median follow-up of 8.3 years	Physically active groups were less likely to develop LVH than sedentary groups. BP declined in physically active patients and slightly increased in their sedentary peers.
Cassidy et al. [47]	Randomized controlled trial	28 patients with type 2 diabetes 61 ± 9 vs. 59 ± 9 years of age 12-week HIIT or standard care	HIIT improved LV wall mass and stroke volume. Early diastolic filling rates increased, and peak torsion decreased in the treatment group.
Otten et al. [48]	Randomized controlled trial	22 overweight and obese subjects with type 2 diabetes 61 (58–66) vs. 59 (52–64) years of age 12-week PD-EX vs. PD and standard care	Significant decreases in the LV mass-to-EDV ratio was observed in the PD-EX group. LVEDV and stroke volume increased significantly only in the PD-EX group.
Gulsin et al. [49]	Randomized controlled trial	87 patients with type 2 diabetes and 36 matched controls 50.5 ± 6.5 vs. 48.6 ± 6.2 years of age 12-week supervised aerobic exercise training vs. low-energy MRP diet vs. routine care	Supervised aerobic exercise training program improved diastolic function in the absence of any major effects on LV remodeling, perfusion, or aortic stiffening. MRP resulted in weight loss and improved blood pressure, glycemia, LV mass/volume, and aortic stiffness but not diastolic function.
Kamimura et al. [51]	Retrospective cohort study	1300 African Americans with preserved LVEF (>50%) 63 (57–69) years of age physical activity was calculated as 3*heavy activity hours + 2*moderate activity hours + slight activity hours/day	Higher physical activity index was independently associated with lower LV mass. Higher physical activity index was associated with lower LV mass index more in obese or hypertensive participants compared with non-obese or non-hypertensive participants.
Himeno et al. [52]	Prospective cohort study	11 obese and hypertensive patients and 11 obese and normotensive patients 37 ± 11 vs. 35 ± 7 years of age 12-week weight-reduction program consisted of mild exercise and mild hypocaloric intake	Systolic, diastolic, and mean BP were significantly reduced only in the hypertensive group. LV mass was significantly reduced both among hypertensive and normotensive obese patients.

### 3.4. In Patients with Coronary Artery Disease

LV remodeling following acute myocardial infraction (AMI) is a complex process characterized by fibroblast proliferation, collagen deposition, scar formation, as well as ventricular expansion, resulting in LV dysfunction and HF, thus negatively affecting long-term prognosis in these patients [55]. The beneficial effect of exercise training on cardiovascular mortality and morbidity, functional capacity, and quality of life in patients with AMI is quite well documented so far [56]. Plenty of studies demonstrated that exercise training affects favorably LVH and LV remodeling. Interestingly, exercise training might reverse LVH to a normal status or at least undergo concentric remodeling. Moreover, there is evidence that training, especially aerobic, improves LVEF and decreases end-diastolic volume (EDV) and end-systolic volume (ESV) [57]. According to a large meta-analysis, exercise training leads to an increase in LVEF, as well as reduction in ESV and EDV in clinically stable post-MI patients [58]. Of note, the greatest benefits in LVEF, ESV, and EDV are occurring when exercise training starts earlier following MI and lasts longer than 3 months, with each week of training delay requiring one additional month of training to achieve the same level of improvement in LV remodeling and the comparable reduction in volumes. Moreover, a decrease in plasma NT-pro-BNP and an increase in peak early mitral flow velocity have been also observed post-training [59]. Additionally, there is evidence that systematic exercise and participation in cardiac rehabilitation programs may significantly improve the cardiorespiratory function, exercise ability, and quality of life in patients with ischemic and nonobstructive coronary arteries (INOCA) [60]. Nevertheless, prolonged endurance exercise training seems to also have detrimental effects on LV systolic and diastolic function.

Exercise training started early after STEMI reduces stress-induced hypoperfusion and improves LV function and contractility. Exercise-induced changes in myocardial perfusion and function is associated with the absence of unfavorable LV remodeling and with an improvement of cardiovascular functional capacity [61]. The beneficial effect of exercise on LV remodeling and cardiopulmonary rehabilitation in LV dysfunction among post-MI patients is also verified by Zhang et al. In this large meta-analysis, it was reported that the greatest benefit of exercise on LV remodeling and cardiopulmonary capacity rehabilitation, as assessed by peak oxygen uptake (VO2), was observed when exercise was initiated in the acute phase after MI, without an increase in the incidence of MACEs [62]. Indeed, during the healing phase after acute MI, the beneficial effects of exercise training on LVEF, LVESD, and peak VO2 weakened compared to the acute phase. Even HIIT seems to improve exercise capacity and quality of life without any detrimental effects on LV remodeling [58,63]. These data imply that secondary prevention along with cardiac rehabilitation programs should be initiated early to achieve the maximal anti-remodeling benefit.

### 3.5. In patients with Heart Failure

Obesity and physical inactivity are considered major lifestyle risk factors for the development and progression of HFpEF [34]. Indeed, low fitness has been associated with a greater risk of HFpEF than HFrEF, whereas patients with HFpEF demonstrate impaired peak VO2 and cardiorespiratory fitness (CRF), which encompasses exercise intolerance, when compared to healthy individuals, deteriorating substantially their prognosis [64].

Physical activity and fitness substantially reduce the risk of developing HF and improve the cardiovascular prognosis among patients with established HF [65]. Interestingly, for every 1-MET improvement in functional capacity, the risk of HF is reduced by 17% [66]. Participation in exercise-based cardiac rehabilitation programs seems to increase exercise capacity by up to 25% and improves the New York Heart Association’s (NYHA) functional status, as well as LV remodeling and hypertrophy. Of note, there is evidence that the beneficial effect of training programs on symptoms, CRF, left ventricular diastolic and systolic function, as well as quality of life and HF-related hospitalizations are also apparent among patients with HFpEF and HFrEF [67]. Moreover, it is also reported that even endurance training reverses adverse cardiac remodeling, and reduces ESV and EDV, thus improving both systolic and diastolic dysfunction in patients with HFrEF [68]. Similarly, high-intensity training beneficially affects exercise capacity and quality of life, with no detrimental changes in LV remodeling among patients with HFrEF [69]. As a result, patients with higher levels of physical activity experience less adverse cardiac events [70].

According to the current literature, exercise training has been associated with a substantial improvement in LV diastolic function [65]. Moreover, it has been reported that exercise reduces LV volumes, which are surrogate markers of LV concentric remodeling or LVH, in patients with HFpEF, whereas exercise training is also correlated with improved measures of CRF. These data demonstrate the pathophysiologic role of LV concentric remodeling contributing to impaired CRF and exercise intolerance in patients with HFpEF and imply that the exercise-meditated improvement in LV remodeling may improve CRF in patients with HFpEF, thus providing novel therapeutical implications [71].

## 4. Pathophysiological Mechanisms of Exercise-Mediated Favorable Cardiovascular Outcomes

One possible explanation for the favorable effect of exercise training on LV remodeling is the reduction in blood pressure [72]. Afterload reduction to LV mediated by lowering blood pressure has been proposed as a possible mechanism leading to the regression of LVH and concentric remodeling [41]. Additionally, favorable effects of exercise training on the cardiovascular system are mainly attributed to changes in vascular function, cardiac energy metabolism, and autonomic balance, including improvement in endothelial dysfunction, arterial and LV stiffness, myocyte calcium handling, mitochondrial function, systolic and diastolic wall stress, cardiac output, and oxygen extraction in the active skeletal muscles [73,74]. These changes are also accompanied by an improvement in cardiac-related biomarkers. Favorable effects include reductions in triglycerides, total cholesterol, low-density lipoprotein, glucose, and insulin and an elevation in high-density lipoprotein following exercise intervention [13]. The strongest evidence indicates that exercise is favorable for the reduction in glucose and cholesterol levels among obese patients and a reduction in insulin regardless of population.

Moreover, increased vasculogenesis through the activation of endothelial progenitor cells ameliorates ischemia and reperfusion injury. Exercise modifies shear stress conditions and further modulates the bioavailability of endothelial nitric oxide (NO), which limits oxidative burden, thus promoting vasodilation and improving vascular endothelial function [39]. Specifically, improvement of endothelium-dependent relaxation is mainly regulated by decreased NO scavenging by reactive oxygen species (ROS) and increased vascular NO production and release. Furthermore, exercise is related to decreased chronic, low-grade inflammation, increasing the release of anti-inflammatory peptides and reducing the production of pro-inflammatory cytokines [75].

Moreover, an exercise-mediated reduction in myocardial collagen has been also described. So, another possible mechanism of an exercise-mediated improvement in LV dysfunction is the upregulation of miRNA-29, which has been associated with a drop in collagen gene expression [76]. Moreover, exercise training reduces total peripheral resistance [39], increases parasympathetic activity, and restores arterial baroreflex sensitivity (BRS), thus ameliorating LVEF and heart rate variability [77]. Nevertheless, many pathophysiological mechanisms responsible for exercise-mediated regression of LVH remain still unclear.

## 5. Recommendations and New Perspectives

According to the 2019 American College of Cardiology (ACC) in collaboration with (AHA) Guideline on the Primary Prevention of CVD, in general, healthy individuals should undergo at least 150 min/week of accumulated moderate-intensity physical activity. Otherwise, 75 min/week of vigorous-intensity physical activity to reduce the risk of atherosclerotic CVD [78]. In total agreement to previous recommendations, the European Society of Cardiology (ESC) highlights that additional benefit is achieved by doubling the previous exercise duration, with multiple sessions of exercise carried out throughout the week [79]. More specifically, hypertensive patients should be advised to participate in at least 30 min of moderate-intensity dynamic aerobic exercise (walking, jogging, cycling, or swimming) on 5–7 days per week [80]. Performance of resistance (and especially isometric) exercise on 2–3 days per week can also be advised. However, patients, who are unable to perform the recommended and desirable regular physical activity, should be encouraged to participate in training programs even below the target goal, considering that the exercise-mediated benefit for the cardiovascular system still remains. Therefore, healthcare professionals should offer patient-focused counseling, encourage their patients to participate in well-organized supervised training programs and help them to optimize their physical activity status, considering the favorable effect of exercise on cardiometabolic risk factors and LVH regression [78].

The European Association of Preventive Cardiology Exercise Prescription in Everyday Practice and Rehabilitative Training (EXPERT) tool is an interactive, digital training, and support system, which aims to help sports medicine specialists, family physicians, cardiologists, and other medical specialists prescribe clinically effective and medically safe exercise training programs for patients with established CVD or cardiometabolic risk factors [81]. In further detail, this website application will analyze patient characteristics, the presence of CVD or cardiometabolic risk factors, contaminant medication, or other clinically relevant medical information, so as to generate the most suitable exercise training program for each patient, discouraging certain types of exercise and providing personalized safety advice. Of note, individualized recommendations regarding exercise training intensity, frequency, duration, type, session, as well as additional exercise training modalities will be offered to the patient.

## 6. Conclusions

Improving exercise capacity and optimizing safety of cardiorespiratory programs play a pivotal role in promoting cardiovascular health. Exercise training exerts beneficial changes to LV remodeling, thus improving cardiac function and structure among patients with cardiometabolic risk factors. These favorable effects are mainly attributed to positive hemodynamic changes in vascular function, cardiac energy metabolism, and autonomic balance. This has the potential to be most impactful in primary care health facilities and countries with limited resources. Implementing basic exercise programs might be a cost-effective strategy to prevent and manage CVD among patients with cardiometabolic risk factors, especially in resource-constrained settings. Thus, it is of major importance that a guided, detailed, and personalized exercise prescription is offered by all physicians based on the individual’s behavioral habits and wishes and cardiovascular profile and needs, so as to achieve better treatment, as well as health outcomes.

## Data Availability

Not applicable.
